# NSP6 of SARS-CoV-2 Dually Regulates Autophagic–Lysosomal Degradation

**DOI:** 10.3390/ijms26083699

**Published:** 2025-04-14

**Authors:** Haijiao Zhang, Jianying Chang, Ren Sheng

**Affiliations:** College of Life and Health Science, Northeastern University, Shenyang 110819, China; 1910067@stu.neu.edu.cn (H.Z.); 2201436@stu.neu.edu.cn (J.C.)

**Keywords:** SARS-CoV-2, COVID-19, NSP6, Beclin1, MLN1, autophagy–lysosome degradation

## Abstract

The pandemic of coronavirus disease 2019 (COVID-19), brought about by the severe acute respiratory syndrome coronavirus 2 (SARS-CoV-2), has significantly impacted public health and the economy. A fundamental aspect of addressing this virus lies in elucidating the mechanisms through which it induces disease. Our study reveals that Non-structural protein 6 (NSP6) of SARS-CoV-2 promotes the initiation of autophagy by activating Beclin1. In the later stage of autophagy, however, NSP6 causes a blockage in the autophagy–lysosome degradation via the inhibition of Mucolipin 1 (MLN1). The single nucleotide polymorphism (SNP) L37F in NSP6, which is associated with asymptomatic infection, similarly enhances the initiation of autophagy but displays a reduced ability to impede lysosome-dependent degradation. In summary, we demonstrated the dual-regulation mechanism of NSP6 in autophagy, which may be one of the reasons for targeting cellular autophagy to induce viral pathogenesis. This finding may provide promising new directions for future research and clinical interventions.

## 1. Introduction

Severe acute respiratory syndrome coronavirus 2 (SARS-CoV-2), classified as an enveloped β-coronavirus with a single-stranded positive-sense RNA genome [1,2], emerged in late 2019, leading to a significant number of coronavirus disease 2019 (COVID-19) patients developing pneumonia and potentially progressing to acute respiratory distress syndrome. This syndrome is defined by swift, extensive lung inflammation and a high mortality rate [3]. Extensive research within the scientific community has focused on the replication and pathogenesis of SARS-CoV-2 for the development of vaccines and therapeutics. Nonetheless, the exact process through which SARS-CoV-2 interferes with cellular activities to promote disease development is still not fully understood.

Autophagy serves as a crucial defense mechanism in the host’s response to viral infections [4,5]. It involves encapsulating cytoplasmic components within double-membrane autophagosomes, which are subsequently transported to lysosomes for breakdown [6,7,8]. The degradation process mediated by lysosomes not only aims to break down viral components but also aids in antigen processing and supports adaptive immune responses [9]. Despite this, certain positive-strand RNA viruses, like coronaviruses, have developed strategies to utilize autophagy for their intracellular replication and dissemination [10,11,12]. In the context of viral infections, particularly during the COVID-19 pandemic, numerous studies have emerged regarding the complex molecular network of interplay between autophagy and SARS-CoV-2 [13]. Additionally, the findings suggest that the expression of NSP6 may also obstruct the autophagic–lysosomal degradation pathway, although the precise mechanisms are yet to be fully understood [14].

Given the development of NSP6 mutations during viral transmission and their association with viral pathogenicity, understanding the function of NSP6 in the pathogenesis of COVID-19 is crucial [15,16,17]. Our study reveals that NSP6 promotes the initiation of autophagy by enhancing the phosphorylation of Beclin1. Furthermore, NSP6 can impede the efficient degradation of autophagic lysosomes by binding to the MLN1 protein. We further demonstrate that the SNP L37F in NSP6, which is linked to asymptomatic infections [15], effectively promotes the initiation of autophagy by regulating Beclin1. However, this mutant has a reduced interaction for the MLN1 and shows decreased capability to suppress the autophagic–lysosomal degradation. This may be one reason for the strain’s weakness. Overall, we have discovered that NSP6 can dually regulate the autophagy process. These findings offer new insights into the mechanisms by which viral proteins regulate autophagosome–lysosome degradation.

## 2. Results

### 2.1. NSP6 and Its SNP L37F Promote the Initiation of Autophagy

The impact of SARS-CoV-2-encoded NSP6 and its SNP L37F on autophagy was investigated using the RFP-GFP-LC3 system. This technique involves monitoring the fusion process between autophagosomes and lysosomes, enabling the evaluation of the autophagic process [14,18]. Since some studies have indicated that adding a tag to the C-terminus of NSP6 can affect its function, the NSP6 and L37F we constructed both had N-terminal protein tags [19].

**Figure 1:**
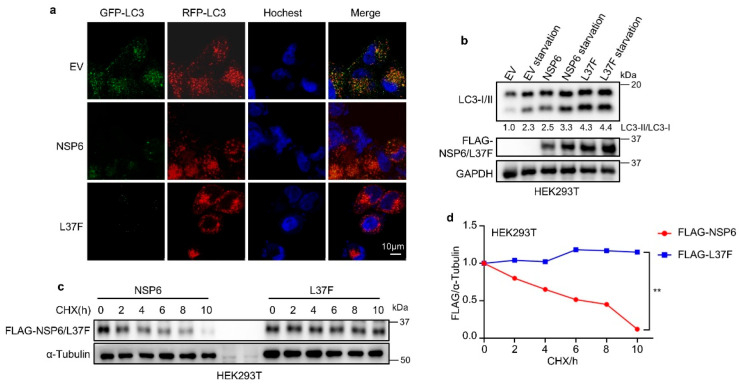
NSP6 and its SNP L37F promote the initiation of autophagy. (**a**) HEK293T cells expressing NSP6 or L37F contain more red-only LC3 puncta. (**b**) Immunoblotting assays revealed higher levels of LC3-II/LC3-I in HEK293T cells expressing NSP6 or L37F under normal serum culture conditions or during starvation treatment. (**c**,**d**) The CHX-blocking assay demonstrated that L37F exhibited superior protein stability compared to the wild type, with quantification results presented in (**d**). The error bar means ± SD, by Student’s two-tailed *t*-test. **, *p* < 0.01.

Cells expressing NSP6 or L37F exhibited more red-only LC3 puncta (Figure 1a), and immunoblotting revealed an increased ratio of LC3-II/LC3-I with NSP6 or L37F expression (Figure 1b), indicating the acidification of autophagic structures and the enhanced initiation of autophagy. Moreover, after the expression of L37F, the ratio of LC3-II to LC3-I exhibited an increasing trend compared to wild type (Figure 1b). A cycloheximide-blocking (CHX) experiment was conducted to assess the protein stability of NSP6 and L37F, revealing that L37F exhibited superior protein stability (Figure 1c,d), potentially contributing to its enhanced ability to induce autophagy initiation.

### 2.2. NSP6 and L37F Can Interact with the ATG14/Beclin1/VPS34 Complex

**Figure 2:**
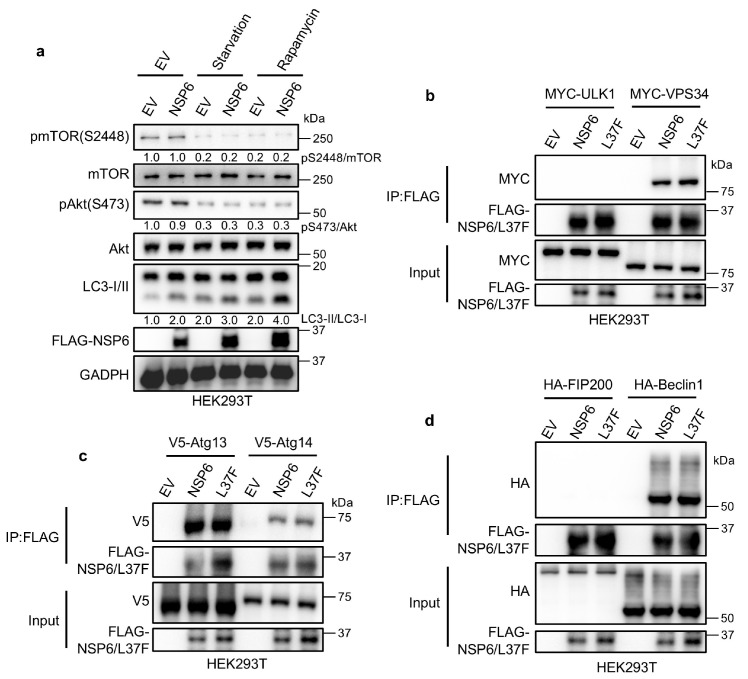
NSP6 and L37F can interact with the ATG14/Beclin1/VPS34 complex. (**a**) Immunoblotting assays revealed that HEK293T cells expressing NSP6 did not exhibit any alteration in Akt s473 or mTOR s2448 phosphorylation sites but an increase in the LC3-II/LC3-I ratio under normal culture conditions, during starvation treatment, or upon addition of rapamycin (1 μM). (**b**–**d**) Co-IP assays demonstrated that NSP6 and L37F interacted with all components of the ATG14/Beclin1/VPS34 complex, along with ATG13. The empty vector (EV) served as a control to exclude nonspecific effects in the experiment.

Autophagy is initiated through a set of interactions involving the mTOR kinase and the ULK1 protein complex [20]. To determine the specific mechanisms by which NSP6 and its SNP L37F impact the autophagy-inducing capabilities, we initially investigated their influence on the upstream regulation of autophagy. When we inhibited mTOR activity through serum starvation [21,22] or rapamycin treatment [23,24], the expression of NSP6 led to an increased LC3-II/LC3-I ratio, without affecting the phosphorylation of Akt and mTOR (Figure 2a). This suggests that NSP6 and L37F do not regulate autophagy through the upstream pathway.

Upon autophagy induction, the complexes ATG13/FIP200/ULK1 and ATG14/Beclin1/VPS34 work in a step-by-step manner to target and generate ER subdomains known as omegasomes [25]. We analyzed the expression of various markers labeling autophagic structures at different stages. Through co-immunoprecipitation (co-IP), we can see that NSP6 could interact with all components of the ATG14/Beclin1/VPS34 complex and ATG13 (Figure 2b,d). This led us to speculate that NSP6 and L37F might regulate autophagy by modulating the ATG14/Beclin1/VPS34 complex.

### 2.3. NSP6 and L37F Enhance the Initiation of Autophagy by Activating Beclin1

**Figure 3:**
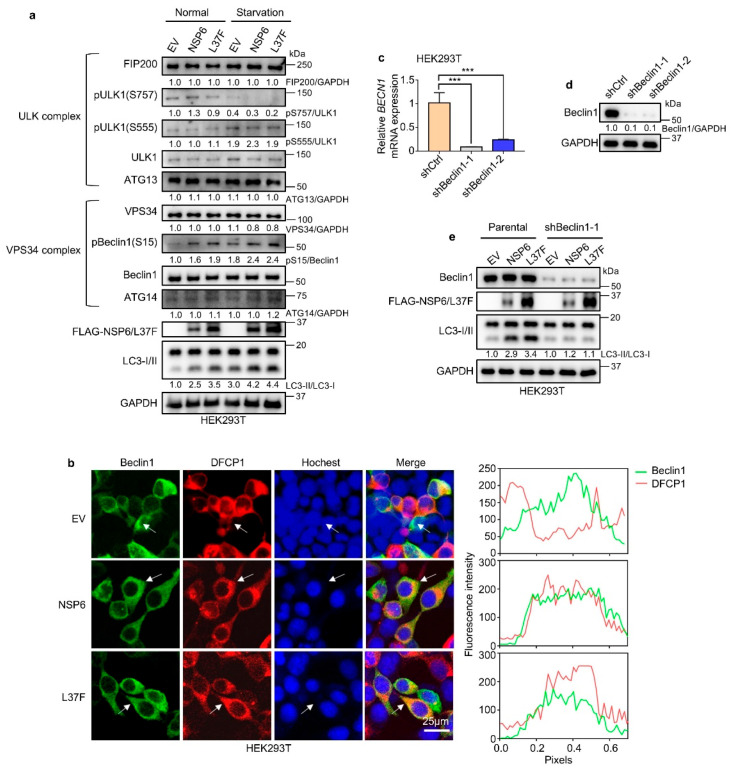
NSP6 and L37F enhance the initiation of autophagy by activating Beclin1. (**a**) Immunoblotting assays demonstrated an increase in phosphorylation at the Beclin1 S15 site upon expression of NSP6 or L37F, both under normal culture conditions and during serum starvation. (**b**) Immunofluorescence results indicated that NSP6 and L37F enhanced the co-localization of Beclin1 and DFCP1. The quantification location in the co-localization quantitative image on the right is indicated by the arrows in the fluorescence image on the left. (**c**) Quantification of qPCR confirmed the knockdown of Beclin1 in HEK293T cells. The error bar means ± SD, n = 3, by a one-way ANOVA. ***, *p* < 0.001. (**d**) The knockdown of Beclin1 in HEK293T cells was confirmed through immunoblotting. (**e**) Immunoblotting assays indicated that NSP6 and L37F were no longer able to enhance the LC3-II/LC3-I ratio following Beclin1 knockdown.

To further investigate the specific mechanism by which NSP6 regulates autophagy, immunoblotting was conducted to analyze the complexes ATG13/FIP200/ULK1 and ATG14/Beclin1/VPS34 (Figure 3a). The results showed no significant change in the ATG13/FIP200/ULK1 complex post NSP6 expression, while phosphorylated Beclin1 was notably increased in the ATG14/Beclin1/VPS34 complex, both in normal serum culture and under starvation conditions. This suggests that NSP6 and L37F may enhance the initiation of autophagy by modulating Beclin1.

During autophagy, DFCP1 is primarily localized in the omegasome and, in conjunction with Beclin1 or other factors, regulates the process [26,27]. The fluorescence co-localization analysis revealed an enhancement in the co-localization of Beclin1 and DFCP1 following NSP6 or L37F expression (Figure 3b). Knocking down Beclin1 resulted in the loss of autophagy-regulating abilities by NSP6 and L37F (Figure 3c–e). These findings support the hypothesis that NSP6 and L37F impact the ATG14/Beclin1/VPS34 complex by activating Beclin1, ultimately leading to the enhanced initiation of autophagy.

### 2.4. NSP6 Inhibits Autophagic–Lysosomal Degradation Through Inhibition of MLN1

**Figure 4:**
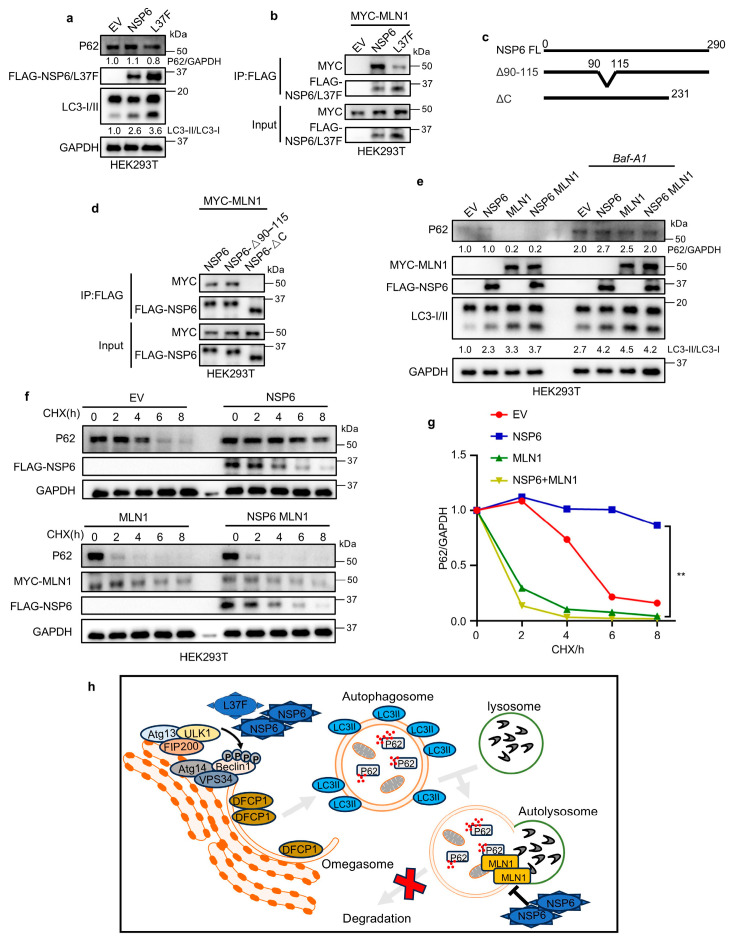
NSP6 inhibits autophagic–lysosomal degradation through inhibition of MLN1. (**a**) Immunoblotting assay demonstrates that NSP6 inhibit lysosomal degradation of P62, and the ability of L37F to do so is diminished. (**b**) Co-IP assay reveals that NSP6 and L37F interact with MLN1, with L37F showing relatively weaker binding. (**c**) Schematic diagram of the NSP6 truncation mutation. (**d**) MLN1 interacts with the amino acid region at positions 231-290 of NSP6. (**e**) No degradation of P62 was observed following treatment with *Baf-A1* even in the case of MLN1 expression. (**f**,**g**) Immunoblotting assay and CHX-blocking assay showing MLN1 can compensate for the inhibition of P62 degradation caused by NSP6 with quantification results presented in (**g**). The error bar means ± SD, by Student’s two-tailed *t*-test. **, *p* < 0.01. (**h**) Working model of NSP6 and L37F regulated the autophagic–lysosomal degradation pathway.

The levels of protein P62, a specific substrate for autophagy, can be utilized to monitor changes in autophagy levels. An increase ratio of LC3-II/LC3-I along with a decrease in P62 indicates a smooth autophagy flux [28]. However, our observations revealed that despite an increase ratio of LC3-II/LC3-I, P62 levels did not decrease appropriately following the expression of NSP6 (Figure 4a), suggesting a blockage in the autophagic–lysosomal degradation pathway. This implies that although NSP6 may enhance the initiation of autophagy, the proteins cannot be efficiently degraded. Interestingly, our study revealed a decrease in P62 levels following the expression of L37F (Figure 4a). Through a review of studies, we identified a protein known as Mucolipin 1 (MLN1). The degradation of autophagosomes and lysosomes does not occur immediately upon fusion. Notably, the deletion of MLN1 leads to increased autophagy while simultaneously decreasing autophagosome degradation. This results in inefficient autophagic processes and the accumulation of ubiquitinated proteins [29,30]. The Co-IP showed that NSP6 and L37F could interact with MLN1 (Figure 4b). In addition, our results suggest that the relatively weak interaction between L37F and MLN1 may have weakened its inhibition of autophagic–lysosomal degradation and contributed to the degradation of P62 (Figure 4b).

Previous studies have highlighted the importance of amino acid regions at positions 91–112 and 231–290 of NSP6 for its structure, leading us to construct truncated mutants [31] (Figure 4c). Our findings indicated that MLN1 primarily interacted with the amino acid region at positions 231-290 of NSP6 (Figure 4d). We noticed that the interaction between L37F and MLN1 was weakened, despite the L37 locus not being situated in that region. Our hypothesis is that a structural alteration in L37F [32] might be responsible for this outcome. However, the specific reasons are not clear, and further exploration is needed in the future.

Furthermore, we observed that P62 degradation occurred upon the expression of MLN1, compensating for the inability of autophagic lysosomes to degrade proteins due to NSP6 (Figure 4e). Subsequent treatment with bafilomycin *A1*(*Baf-A1*) [33], a late autophagy inhibitor, prevented the degradation of P62 even in the case of MLN1 expression (Figure 4e), indicating that the process indeed followed the autophagic–lysosomal degradation pathway. The same conclusion was obtained using the CHX blocking assay to determine P62 protein stability (Figure 4f,g). Taken together, our results suggest that NSP6 may bind to and inhibit MLN1, consequently hindering the effective degradation of proteins by the autophagy–lysosome pathway.

Therefore, our research reveals that NSP6 regulates the autophagy pathway through a dual regulatory mechanism. First, the expression of NSP6 and L37F elevates Beclin1 phosphorylation levels, thereby enhancing the initiation of autophagy (Figure 4h). Additionally, NSP6 can bind to MLN1, inhibiting the degradation of P62, which results in the blockage of autophagic flux (Figure 4h). The SNP L37F in NSP6 has an attenuated interaction with MLN1 and shows some degradation of P62 following enhanced autophagy.

## 3. Discussion

Since its emergence, COVID-19 has rapidly spread worldwide. Therefore, understanding the pathogenesis of SARS-CoV-2 remains an important problem for the scientific community. Our results suggest that SARS-CoV-2 may utilize NSP6 to inhibit autophagic-lysosomal degradation, thereby isolating virus-associated proteins within the cell. This isolation prevents recognition and degradation by the body’s defense system, contributing to viral amplification and propagation. The virulence and pathogenicity of asymptomatic COVID-19 are still a mystery. Asymptomatic infection has been linked to the SNP L37F in NSP6 [15,34]. We speculate that COVID-19, through this mutation, weakens its inhibition of autophagic–lysosomal degradation, thereby facilitating the degradation of relevant viral components. This may elucidate the mechanism by which the virus becomes a weaker strain following the emergence of L37F.

We also found that L37F enhanced the protein stability of NSP6. The L37 position is located at the N-terminal region of the NSP6 protein, near the junction between the first transmembrane helix (TM1) and the luminal loop [15]. After mutation, the introduction of a larger aromatic group may cause changes in the local conformation and properties of the protein. Studies have shown that the L37F mutation exhibits significant conformational differences compared to the wild type [32], resulting in increased rigidity of the NSP6 local structure (first α-helix) [15]. This increased rigidity may, to some extent, affect the degradation pathway of NSP6. Other research has indicated that the K61 ubiquitination of NSP6 aids in recruiting downstream signaling proteins to activate NF-κB; however, the pathogenicity of the L37F mutant strain is reduced [15]. Therefore, we might speculate that the L37F mutation leads to an increase in protein levels by affecting the ubiquitination process. Generally, ubiquitination occurs on the lysine (K) residues of proteins, and the structural changes caused by the L37F mutation may prevent the E3 ubiquitin ligase TRIM13 from connecting to the K61 residue (or other potential ubiquitination sites) or may somehow reduce the degree of ubiquitination modification of the protein [35]. However, these are all hypotheses that require further detailed investigation in the future.

Notably, several publications have reported multiple roles of NSP6 in the pathogenesis of SARS-CoV-2. For instance, NSP6 can regulate pyroptosis and degrade STING1 by triggering endoplasmic reticulum stress-induced autophagy. Additionally, it plays a role in the biogenesis of SARS-CoV-2 replicating organelles and is crucial for evading type I interferons [19,34,36,37,38]. It has also been reported that NSP6 enhances the initiation of autophagy, although the precise mechanism remains to be elucidated in detail [13]. A study suggests that NSP6 inhibits lysosomal acidification by targeting ATP6AP1, thereby hindering autophagic flux [34]. We speculate that NSP6 influences autophagic–lysosomal degradation through a combination of factors. Therefore, we recommend that P3 laboratories equipped to conduct viral experiments integrate in vivo studies to systematically and comprehensively explore this phenomenon. Such investigations will not only clarify the specific and comprehensive functions of NSP6 in COVID-19 but may also illuminate the pathogenesis of a range of coronaviruses.

While progress has been made in vaccine research and popularization, the treatment of COVID-19 continues to be a complex issue [39]. Our findings suggest that targeting the interaction between NSP6 and the ATG14/Beclin1/VPS34 complex or MLN1 could serve as a promising therapeutic avenue in the treatment of COVID-19. This approach offers innovative strategies for treating novel coronaviruses and other related strains, holding considerable potential for clinical application.

## 4. Materials and Methods

### 4.1. Key Materials

All key materials are listed in Table S1 in the Supplementary Materials.

### 4.2. Cell Lines

The cell lines, both the original and genetically altered, were cultured in DMEM medium supplemented with 10% FBS and 100 mg/mL of solution containing penicillin, streptomycin, and glutamine. These cultures were maintained in incubators with 5% CO_2_ at 37 °C. Further information regarding the cell lines can be found in Table S1.

### 4.3. Clones and Constructs

All plasmids are listed in Table S1 and were transferred into *E. coli* NEB^®^5α strain for replication. Following that, the plasmids were isolated utilizing the OMEGA Endo-free Plasmid Mini Kit (OMEGA, Norcross, GA, USA), and the concentrations for each plasmid were measured using the Thermo Nanodrop 2000 (Thermo Fisher, Waltham, MA, USA).

### 4.4. Antibodies and Immunoblotting

Cells underwent three washes with cold PBS, followed by lysis on ice for 10 min in a lysis buffer supplemented with a protease inhibitor. The resulting lysates were mixed with an SDS loading buffer and heated to 95 °C for 5 min to prepare protein samples. These protein samples were then separated on 6–10% SDS-PAGE gels and transferred to PVDF membranes (Millipore, Burlington, MA, USA). The membranes were subsequently blocked with 3% BSA diluted in TBS-T buffer and incubated overnight at 4 °C with primary antibodies at a 1:1000 dilution. After three 10-min washes with TBS-T, the membranes were incubated at room temperature for 45 min with secondary antibodies at a 1:5000 dilution. Before exposure, the membranes underwent another three 10-min washes with the TBS-T buffer. Detection was carried out using the Tanon chemiluminescent substrate kit in conjunction with the Tanon 5200 Chemiluminescence Imaging System. ImageJ (v. 1.52a, https://imagej.net/ij/) was used to quantify protein levels. For detailed information on all the antibodies utilized, please refer to Table S1.

### 4.5. Cycloheximide-Blocking Assay

Cells were cultured in 24-well plates and treated with cycloheximide (CHX) at a concentration of 300 μg/mL for the specified durations. Following a wash with PBS, the cells were then prepared for immunoblotting according to the previously mentioned protocol.

### 4.6. Cell Transfection

Neofect was used as the transfection reagent in the transfection protocol. For transient cDNA transfection, Neofect was utilized in accordance with the instructions provided. Plasmids were diluted with DMEM and mixed with Neofect. After incubating at room temperature for 20 min, the mixtures were applied to HEK293T cells via the growth medium. Fresh medium was substituted after a 24-h incubation period.

For experiments using 24-well plates, cells were transfected with a total plasmid amount of 500 ng per well. For experiments using 12-well plates, cells were transfected with a total plasmid amount of 1 μg per well. In co-transfection experiments requiring simultaneous expression of two plasmids, half of the total plasmid amount was allocated to each plasmid (e.g., 250 ng per plasmid in 24-well plates or 500 ng per plasmid in 12-well plates).

### 4.7. Lentivirus Production and Infection

The process of Lentivirus production included the co-transfection of the psPAX2 vector (Addgene, Watertown, MA, USA), pCMV-VSV-G (Addgene), and custom pLKO plasmids at a 5:1:5 ratio by weight within HEK293T cells. The lentivirus-containing medium was then collected and utilized for transduction in HEK293T cells.

For lentiviral infection, 0.5–1 mL of lentivirus-containing medium was combined with 1 mL of fresh medium supplemented with polybrene (Santa Cruz Biotech, 1:1000, Santa Cruz, CA, USA). Following a 48-h incubation period, cells were subjected to resistance selection by replacing the medium with puromycin (Invivogen, San Diego, CA, USA).

### 4.8. Immunofluorescence Co-Localization Assay

Cells expressing either control plasmids, NSP6, or L37F were plated on glass coverslips in 12-well plates and fixed with 4% paraformaldehyde (PFA) in PBS at 37 °C for 15 min. Subsequently, they were treated with 0.5% Triton X-100 in PBS for 30 min at room temperature, followed by an incubation with anti-Beclin1 antibody (1:400 in buffer) for 12 to 16 h at 4 °C. After two washes with cold PBS, the cells were incubated with a secondary antibody (Invitrogen, 1:500) for 30 min. DFCP1 itself was tagged with red fluorescence. Finally, fluorescence microscopy images were acquired using a Leica TCS SP8 system.

### 4.9. Reverse Transcription and Quantitative Real-Time PCR (RT-qPCR)

RNA was extracted from cells using the UNlQ-10 Column Trizol Total RNA Isolation Kit (Sangon Biotech, Shanghai, China) for RT-qPCR analysis. The isolated RNA was then transcribed into cDNA using the MonScript™ RTIII All-in-One Mix (Monad, Wuhan, China) as per the manufacturer’s protocol. For the quantitative RT-PCR phase, the MonAmp™ ChemoHS qPCR Mix (Monad) was utilized. The primer sequences were designed based on the reliable primer bank provided by Massachusetts General Hospital (available at https://pga.mgh.harvard.edu/primerbank/index.html, accessed on 5 May 2024), and these sequences are detailed in Table S1. Each experiment was repeated three times. To achieve accurate quantification of gene expression, the data were normalized to the levels of the housekeeping gene *GAPDH*.

### 4.10. FBS Starvation and Rapamycin Treatment Assay

After reaching 40% to 60% confluence in 24-well plates, the cells were washed three times with PBS to remove fetal bovine serum (FBS) from the cell surface. Subsequently, FBS was removed for a 24-h period. For the rapamycin treatment assay, rapamycin (1 μM) was added 24 h prior to sample collection.

### 4.11. Statistics

The quantification of immunoblotting and immunofluorescence co-localization was carried out utilizing ImageJ. Statistical analyses were conducted employing GraphPad Prism 8.0 software (https://www.graphpad.com). Protein expression values were normalized to levels of GAPDH or tubulin. A two-tailed Student’s *t*-test was employed for statistical analysis. Gene expression values were normalized to the level of *GAPDH*. The 2^−ΔΔCt^ method was used to analyze the data. The experimental findings are presented as the mean ± SD, with 3 replicates (*n* = 3), and a statistical analysis was performed utilizing a one-way ANOVA.

## Data Availability

The original contributions presented in this study are included in the article/Supplementary Materials. Further inquiries can be directed to the corresponding author.

## Supplementary Materials

The following supporting information can be downloaded at: https://www.mdpi.com/article/10.3390/ijms26083699/s1.
