# Supplementary material for: NSP6 of SARS-CoV-2 Dually Regulates Autophagic–Lysosomal Degradation
Source: Int J Mol Sci. 2025 Apr 14;26(8):3699. doi: 10.3390/ijms26083699 (PMC12028300; doi:10.3390/ijms26083699)
Supplement: Supplementary file 1 [file ijms-26-03699-s001.zip › ijms-3575535-supplementary.pdf]

Table S1. Key resources table

REAGENT or RESOURCE	Source	Identifier
**Antibodies**		
LC3A/B (D3U4C) Rabbit mAb	Cell Signaling Technology	#12741
DYKDDDDK Tag (D6W5B) Rabbit mAb	Cell Signaling Technology	#14793
HA-Tag (C29F4) Rabbit mAb	Cell Signaling Technology	#3724
MYC-Tag (9B11) Mouse mAb	Cell Signaling Technology	#2276
V5-Tag (D3H8Q) Rabbit mAb	Cell Signaling Technology	#13202
Alpha Tubulin Polyclonal antibody	Proteintech	11224-1-AP
GAPDH Polyclonal antibody	Proteintech	10494-1-AP
Goat anti-Rabbit IgG (H+L) Highly Cross-Adsorbed Secondary Antibody, Alexa Fluor™ 488	Invitrogen	A11034
Anti-rabbit IgG, HRP-linked Antibody	Cell Signaling Technology	#7074
mTOR Antibody	Cell Signaling Technology	#2972
Phospho-mTOR (Ser2448) (D9C2) XP® Rabbit mAb	Cell Signaling Technology	#5536
Phospho-Akt (Ser473) (D9E) Rabbit mAb	Cell Signaling Technology	#4060
Akt (pan) (C67E7) Rabbit mAb	Cell Signaling Technology	#4691
Beclin-1 (D40C5) Rabbit mAb	Cell Signaling Technology	#3495
ULK1 (D8H5) Rabbit mAb	Cell Signaling Technology	#8054
Phospho-ULK1 (Ser555) (D1H4) Rabbit mAb	Cell Signaling Technology	#5869
Phospho-ULK1 (Ser757) Antibody	Cell Signaling Technology	#6888
Phospho-Beclin-1 (Ser15) (D4B7R) Rabbit mAb	Cell Signaling Technology	#84966
Atg13 (E1Y9V) Rabbit mAb	Cell Signaling Technology	#13468
Atg14 (D1A1N) Rabbit mAb	Cell Signaling Technology	#96752
SQSTM1/p62 Antibody	Cell Signaling Technology	#5114
PI3 Kinase Class III (D9A5) Rabbit mAb	Cell Signaling Technology	#4263
FIP200 (D10D11) Rabbit mAb	Cell Signaling Technology	#12436
**Bacterial strain**		
E. coli NEB® 5-alpha	New England Biolabs (NEB)	C2987H
**Commercial chemicals and biologics/Chemicals**		
4% paraformaldehyde	Beyotime Biotechnology	P0099
Penicillin-Streptomycin-Glutamine (100X)	Gibco	10378016
Puromycin	Invivogen	ant-pr-1
Neofect™ DNA transfection reagent	Neofect	ME201901
Polybrene	Santa Cruz Biotechnology	SC-134220
Rapamycin	Selleck	S1039
Bafilomycin A1 (Baf-A1)	Selleck	S1413
Cycloheximide	Sigma-Aldrich	239763-M
Glycerol	Sangon Biotech	A100854
Glycine	Sangon Biotech	A110167
Phosphate buffer	Sangon Biotech	A610100-0001
Sodium dodecyl sulfate	Sangon Biotech	A600485-0500
Thiazolyl Blue Tetrazolium Bromide	Sigma-Aldrich	M5655

Tris[(1-benzyl-1H-1,2,3-triazol-4-yl) methyl] amine	Sigma-Aldrich	678937
Hoechst	Thermo	1990363
**Critical commercial assays/kits**		
Endo-free Plasmid Mini Kit	Omega	D6950
UNlQ-10 Column Trizol Total RNA Isolation Kit	Sangon Biotech	B511321
MonScript™ RTIII All-in-One Mix with dsDNase	Monad	MR05101M
MonAmp™ ChemoHS qPCR Mix	Monad	MQ00401S
GeneJET Gel Extraction Kit	Thermo	K0692
GeneJET PCR Purification Kit	Thermo	K0702
ChemistarTM High-sig ECL Western Blotting Substrate	Tanon	180-5001
**Experimental models: Cell lines**		
HEK293T	ATCC	CRL-11268 RRID:CVCL_1926
**Oligonucleotides**		
Control-shRNA sequence	This study	AAAAAAAAAAAAAAAAAAAAA
#1-shBeclin1 sequence (Human)	This study	CCACTCTGTGAGGAATGCACAGATA
#2-shBeclin1 sequence (Human)	This study	TATCTGTGCATTCCTCACAGAGTGG
Human *GAPDH*-RT-qPCR primers	This study	F:GGAGCGAGATCCCTCCAAAT R:GGCTGTTGTCATACTTCTCAG
Human *BECN1*-RT-qPCR primers	This study	F:ACCTCAGCCGAAGACTGAAG R:AACAGCGTTTGTAGTTCTGACA
**Recombinant DNA**		
psPAX2 vector	Addgene	#12260
pCMV-VSV-G	Addgene	#8454
myc-ULK1	Addgene	#31961
3xFLAG-CMV10-FIP200	Addgene	#24300
mCherry-DFCP1	Addgene	#86746
Lenti-EF1α-puro FLAG-NSP6	This study	
Lenti-EF1α-puro FLAG-L37F	This study	
Lenti-EF1α-puro MYC-VPS34	This study	
Lenti-EF1α-puro V5-Atg13	This study	
Lenti-EF1α-puro V5-Atg 14	This study	
Lenti-EF1α-puro HA-Beclin1	This study	
Lenti-EF1α-puro MYC-MOLN1	This study	
Lenti-EF1α-puro FLAG-Δ90-115	This study	
Lenti-EF1α-puro FLAG-ΔC	This study	
Lenti-EF1α-puro GFP-RFP-LC3	This study	
pLKO-puro shBeclin1#1	This study	

pLKO-puro shBeclin1#2	This study	
pLKO-puro shControl	This study	
**Software and algorithms**		
Graphpad Prism 8	Graphpad software	https://www.graphpad.com
ImageJ		https://imagej.nih.gov/ij/
**Other**		
PVDF	Millipore	IPVH00010
α-FLAG Agarose Affinity Gel	Sigma-Aldrich	4596
